# Short-Chain Fatty Acids—A Product of the Microbiome and Its Participation in Two-Way Communication on the Microbiome-Host Mammal Line

**DOI:** 10.1007/s13679-023-00503-6

**Published:** 2023-05-19

**Authors:** Oliwia Lange, Monika Proczko-Stepaniak, Adriana Mika

**Affiliations:** 1 Department of Environmental Analysis, University of Gdansk, Wita Stwosza 63, 80-308 Gdansk, Poland; 2 Department of Pharmaceutical Biochemistry, Medical University of Gdansk, Debinki 1, 80-211 Gdansk, Poland; 3 Department of General, Endocrine, and Transplant Surgery, Faculty of Medicine, Medical University of Gdansk, Smoluchowskiego 17, 80-214 Gdansk, Poland

**Keywords:** Short-chain fatty acids, Obesity, Microbiota, Diet, Fat metabolism, Bariatric surgery

## Abstract

***Purpose of Review*:**

The review aims to describe short-chain fatty acids (SCFAs) as metabolites of bacteria, their complex influence on whole-body metabolism, and alterations in the SCFA profile in obesity and after bariatric surgery (BS).

***Recent Findings*:**

The fecal profile of SCFAs in obese patients differs from that of lean patients, as well as their gut microbiota composition. In obese patients, a lower diversity of bacteria is observed, as well as higher concentrations of SCFAs in stool samples. Obesity is now considered a global epidemic and bariatric surgery (BS) is an effective treatment for severe obesity. BS affects the structure and functioning of the digestive system, and also alters gut microbiota and the concentration of fecal SCFAs. Generally, after BS, SCFA levels are lower but levels of branched short-chain fatty acids (BSCFAs) are elevated, the effect of which is not fully understood. Moreover, changes in the profile of circulating SCFAs are little known and this is an area for further research.

***Summary*:**

Obesity seems to be inherently associated with changes in the SCFA profile. It is necessary to better understand the impact of BS on microbiota and the metabolome in both feces and blood as only a small percentage of SCFAs are excreted. Further research may allow the development of a personalized therapeutic approach to the BS patient in terms of diet and prebiotic intervention.

## Introduction

**Fig. 1:**
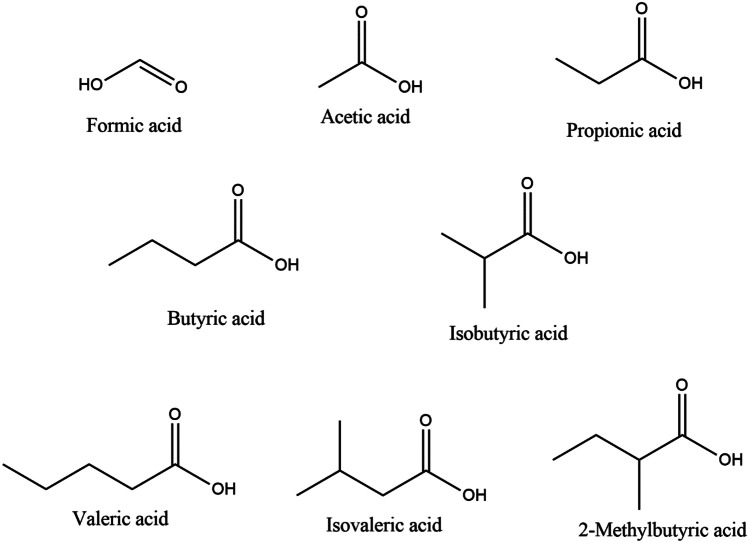
Structure of short-chain fatty acids

**Fig. 2:**
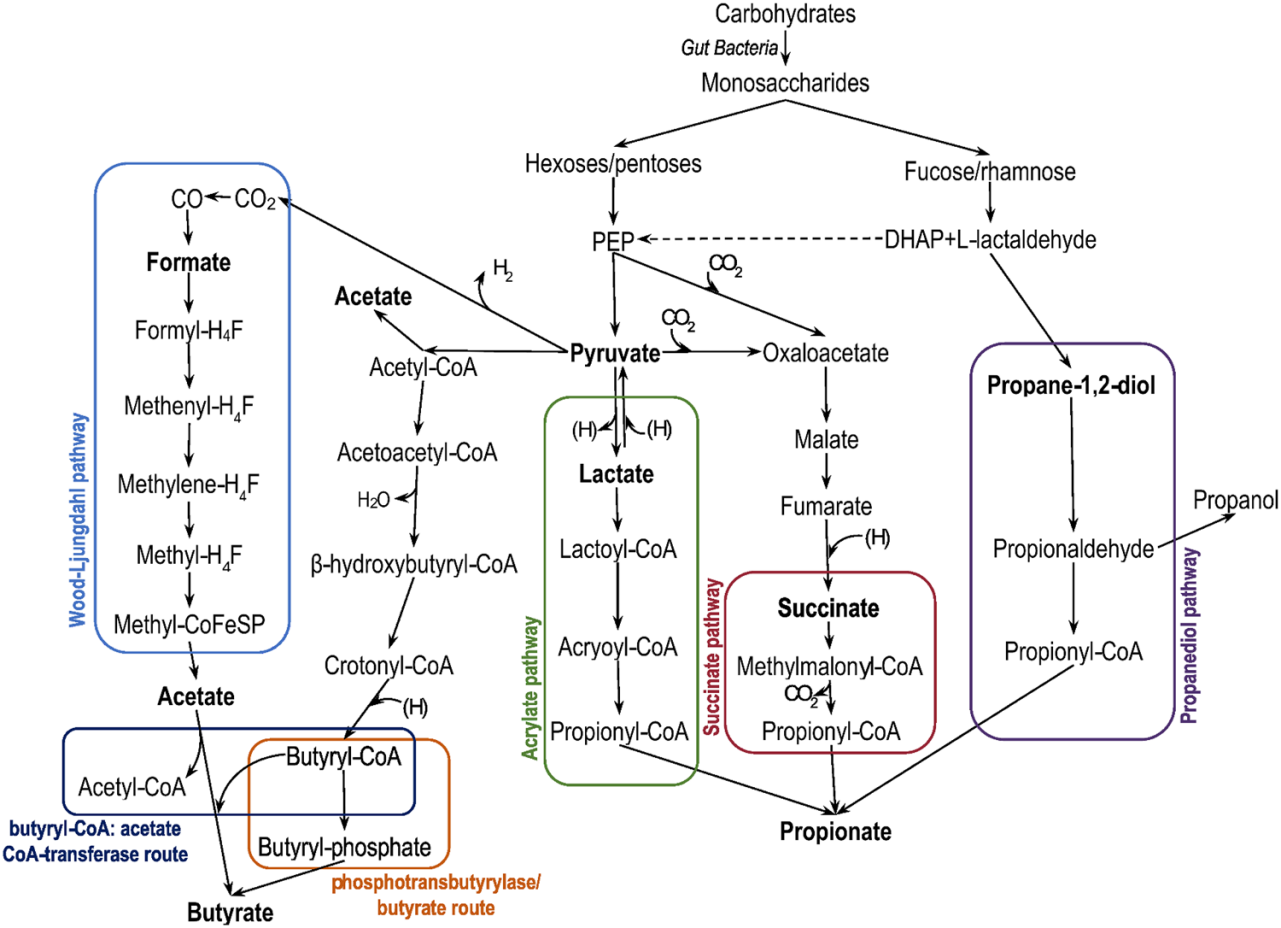
Pathways for the synthesis of SCFAs

Short-chain fatty acids (SCFAs) are organic linear carboxylic acids with fewer than six carbons in the chain (Fig. 1) [1]. Predominantly, SCFAs are produced by intestinal fermentation due to the lack of enzymes to degrade the majority of dietary fibers in the human body [2]. The most abundant are acetic acid (C2), propionic acid (C3), and butyric acid (C4) in an approximate molar ratio of 60:20:20, respectively, in the colon and stool [2, 3]. Gut bacteria utilize different types of non-digestible carbohydrates: plant cell-wall polysaccharides, oligosaccharides, resistant starches. The average intake of fiber in the western diet is about 20–25 g daily [2]. If fermentable fiber supplies decrease, microbes begin metabolizing amino acids or proteins as an alternative. Branched amino acids, such as leucine, isoleucine, or valine, are fermented to branched short-chain fatty acids (BSCFAs) like isovalerate, isobutyrate, and 2-methylbutyrate [1, 4, 5•, 6]. For this reason, BSCFAs or a ratio of SCFAs/BSCFAs have been proposed as a marker for protein fermentation, the products of which can damage the epithelium in the colon [7]. Aside from their presence in bacterial fermentation, SCFAs are present in plant oil and animal fats [4]. The biosynthesis of SCFAs consists of many processes in which a number of bacterial species are involved (Table 1). Most enteric bacteria are producers of acetate, while propionate and butyrate are synthesized by more specific species of gut microbiota. Acetate can be obtained from pyruvate via acetyl-CoA or via the Wood-Ljungdahl pathway, whereas propionate, via the succinate, acrylate, or propanediol pathway. In the Wood-Ljungdahl pathway, which is the major and most efficient pathway of acetate production, CO_2_ is reduced to CO and formic acid or directly to a formyl group, and converted with a methyl group and CoA-SH to acetyl-CoA [1, 2, 6, 8]. Though most hexoses and pentoses enter the succinate pathway, there is also an alternative to produce propionate from amino acids, lactate, or 1,2-propanediol [4]. Aside from being a product of carbohydrates, butyrate can be formed from acetate, lactate, or amino acids via the phosphotransbutyrylase/butyrate kinase or butyryl-CoA:acetate CoA-transferase route [1, 6, 8] (Fig. 2).

**Table 1:** Biosynthesis of SCFAs by gut bacteria [5•, 6]

SCFA	Pathway	Bacteria
Acetate	From pyruvate via acetyl-CoA	Most of the enteric bacteria, e.g., *Akkermansia muciniphila*, *Bacteroides* spp., *Bifidobacterium* spp., *Prevotella* spp., *Ruminococcus* spp.
	Wood-Ljungdahl pathway	*Blautia hydrogenotrophica*, *Clostridium* spp., *Streptococcus* spp.
	Succinate pathway	*Bacteroides* spp., *Phascolarctobacterium succinatutens*, *Dialister* spp., *Veillonella* spp.
Propionate	Acrylate pathway	*Megasphaera elsdenii*,* Coprococcus catus*
	Propanediol pathway	*Salmonella* spp., *Roseburia inulinivorans*, *Ruminococcus obeum*
	Phosphotransbutyrylase/butyrate kinase route	*Firmicutes* spp., *Coprococcus comes*, *Coprococcus eutactus*
Butyrate	From acetate via the butyryl-CoA:acetate CoA-transferase route	*Anaerostipes* spp., *Coprococcus catus*, *Eubacterium rectale*, *Eubacterium hallii*, *Faecalibacterium prausnitzii*, *Roseburia* spp.
	From lactate via the butyryl-CoA:acetate CoA-transferase route	*Anaerostipes* spp., *Eubacterium hallii*, *Faecalibacterium*

Acetate, propionate, and butyrate are almost fully absorbed from the lumen of the gut into the bloodstream. Only < 5% of them are excreted in feces [5•]. The total concentration of SCFAs ranges from 70–140 mmol in the proximal colon to 20–70 mmol in the distal colon [2]. Declining levels of SCFAs are caused by increasing pH and absorption through specific transport proteins. SCFAs, as weak acids, are ionized and require transporters to be absorbed from the colon [4]. There are four transport mechanisms regarding SCFAs: passive diffusion, bicarbonate exchange, sodium-coupled, or monocarboxylate transporters [1, 5•, 9]. SCFAs stimulate sodium and water absorption, which may be utilized as an antidiarrheal factor [9]. Additionally, it is shown that butyrate, as a primary source of energy for colonocytes, is locally absorbed and metabolized by the colonic epithelium [6, 10] effecting low butyrate concentration in portal blood [9]. Both acetate and propionate are drained into the portal vein, but only propionate is metabolized in the liver [4, 5•, 6]. As a result, acetate is the most abundant SCFA in the peripheral circulation, in a venous concentration of 98–143 µmol/l. In turn, the venous concentrations of propionate and butyrate range from 3.8 to 5.4 µmol/l and 0.5 to 3.3 µmol/l, respectively [4, 9]. The content and profile of SCFAs are influenced by diet and the composition of the intestinal microbiota. It is known that microbiota dysbiosis may be caused by many factors, such as inflammation, lifestyle habits, or medication. Based on the increasing incidence of obesity and the inherent association of obesity with changes in the SCFA profile, the aim of this review is to describe the complex effects of SCFAs in whole-body metabolism, examining the recent literature on alterations in the SCFA profile in obesity and after bariatric surgery (BS).

## Functions of SCFAs

### Direct Action on the Gut

#### Gut Barrier

SCFAs play an important role in the gut. Maintaining integrity is essential due to the high density of bacteria in the digestive tract. Otherwise, pathogenic bacteria such as *Chlamydophila pneumoniae* or *Helicobacter pylori* can enter the bloodstream [11]. A key element is tight junctions (TJs), which can be regulated by SCFAs, mostly butyrate, and lead to decreased permeability of the epithelial barrier [12]. SCFAs also stimulate mucus production and create a protective layer between the intestinal lumen and epithelial cells [1, 13]. What is more, SCFAs can stimulate the secretion of antimicrobial peptides (AMPs), and AMPs, such as cathelicidin LL-37, α-defensines, β-defensines, and regenerating islet-derived protein 3 γ (REGIIIγ), are believed to be the first line of defense against many pathogens [11, 13]. Moreover, as described above, butyrate is the preferential SCFA taken up by colonocytes for energy production, and butyrate oxidation can provide up to 70% of their energy requirement [2].

#### Gut Hormones

SCFAs can be used as signaling molecules and activate intestinal G protein–coupled receptors (GPCRs), including GRP41, GRP43, and GPR109a, which are involved in the secretion of gut hormones such as peptide YY (PYY) and glucagon-like peptide 1 (GLP-1). These hormones are secreted in response to nutrient ingestion by L-cells in the gut. Both PYY and GLP-1 reduce appetite and energy intake, delay gastric emptying, and promote insulin secretion. Their fasting concentration is shown to be lower in obese compared to lean individuals [14]. The actions of both hormones are beneficial for the treatment of obesity; hence, drugs are formed based on GLP-1 receptor agonists or PYY analogues [15]. It is shown that a high concentration of colon SCFAs leads to elevated levels of plasma PYY and GLP-1 [16]. GPR41 and GPR43 are also known, respectively, as FFAR3 and FFAR2 as part of the fatty acid receptors group (FFARs). The results of their action are described in the section below. Olfr78 is also expressed in the colon but the effects of this receptor are unclear, possibly being involved in the control of energy metabolism [17].

### Influence on Whole-Body Metabolism

#### Energy Source

It is reported that SCFAs can provide about 10% of the daily caloric requirement as a substrate for the citric acid cycle or beta-oxidation in mitochondria, after conversion to acetyl-CoA. Most acetate circulating in the blood is taken up by the liver for energy and as a substrate for the synthesis of cholesterol and long-chain fatty acids, but also the heart, kidneys, and muscles use acetate to produce energy. It is also shown that acetate can be used for energy-producing purposes in astrocytes [5•]. In turn, propionate and its contribution to energy metabolism is not well understood, but it is known to act as a precursor for gluconeogenesis in the liver [2].

#### Signaling Molecules

Acetate, propionate, and butyrate can also be utilized in signaling in processes throughout the body. They activate GPCRs such as GPR41, GPR43, and GPR109a, which are involved in glucose and lipid regulation. Olfr78 is also a GPCR activated by SCFAs. Moreover, SCFAs can modulate biological responses by the direct inhibition of histone deacetylases (HDACs) to regulate gene expression [1, 5•, 8]. What is more, propionate and butyrate are involved in the activation of the peroxisome proliferator–activated receptor γ (PPAR-γ). It is also worth mentioning that AMP-activated protein kinase (AMPKs) is a target for activation by SCFAs, especially in the liver and muscle tissue [2]. All of the listed receptors are present in various cells in the human body (Table 2). There is a growing interest in modulating the activity of these receptors as potential therapeutic targets in many diseases [17].

**Table 2:** Receptors for SCFAs [8, 17]

Receptor	SCFA	Location	Role
GPR43 (FFAR2)	Acetate, propionate, butyrate	Digestive tract epithelial cells, immune system cells, adipocytes in adipose tissue, enteroendocrine cells, pancreatic β-cells	Production of chemokines, cytokines and epithelial cell protection, anti-inflammatory and antitumorigenic, regulation of the size and function of the colonic T_reg_ pool, TNF-α secretion, modulation of immune cell recruitment during inflammatory responses, reduction of lipolysis, reduction of fat accumulation, suppression of the insulin signal, secretion of PYY and GLP-1, enhancement of glucose-stimulated insulin secretion
GPR41 (FFAR3)	Acetate, propionate, butyrate	Large intestine lamina propria cells, spleen cells, lymph nodes, bone marrow, adipocytes, polymorphonuclear leukocytes, peripheral nervous system cells, distal tubules and kidney collecting ducts, pancreatic β-cells, enteroendocrine cells, myeloid dendric cells, thymus	Activation of sympathetic nervous system, PYY and GLP-1 secretion, leptin production, Th2 cell responses, promotion of thymic T_reg_ differentiation, inhibition of glucose-stimulated insulin secretion, intestinal gluconeogenesis
GPR109a (HCA2)	Butyrate, β-hydroxybutyric acids, niacin, ketone bodies	Large intestinal epithelium, macrophages, monocytes, dendritic cells, neutrophils, adipocytes	High-density lipoprotein metabolism, cAMP reduction in adipocytes, DC trafficking, anti-inflammatory, and antitumorigenic
Olfr-87 (OR51E2)	Acetate, propionate	Neurons, enteroendocrine cells, the epithelium of the large intestine, renal arteries, smooth muscles of blood vessels	Blood pressure modulation
PPAR-γ	Propionate, butyrate	Large intestine adenocarcinoma cells	Glucose transport regulation

#### Blood Pressure Regulation

SCFAs are considered to be linked to blood pressure regulation. They can have both hypertensive and hypotensive effects. Binding with Olfr87 causes an increase in blood pressure, while binding with FFAR3 leads to lower blood pressure [11, 18]. Butyrate can also potentially lower diastolic blood pressure through a reduction of inflammation [18].

#### Glucose Metabolism

SCFAs are involved in glucose metabolism via many mechanisms. First of all, SCFA interactions can lead to lowering the plasma glucose level via increased glucose cell intake mediated by FFAR2 and FFAR3. It is shown that propionate can be beneficial for β-cells in the pancreas and activates FFAR2, which leads to increased β-cell mass and enhanced glucose-stimulated insulin release, whereas high levels of acetate are inversely related to insulin levels in serum [11, 12]. Additionally, as mentioned above, gut hormones secreted as a result of the stimulation of SCFAs are also important for glucose metabolism. PYY, known for being the satiety hormone, and GLP-1 are involved in glucose-dependent insulin secretion, suppressing postprandial glucagon secretion and then lowering blood glucose [2, 11, 14, 19]. Increased secretion of PYY and GLP-1 can enhance glucose uptake by the muscles and adipose tissue and, in effect, raise satiety and reduce food intake. Moreover, leptin secretion from adipose tissue can be regulated by SCFAs by activating GPCRs, especially FFAR2 and FFAR3. Leptin is important in maintaining the energy balance by influencing food intake and energy expenditure [5•, 17].

Another mechanism is to upregulate the mRNA expression of GLUT2 glucose transporters, which are important for signaling pathways in enterocytes and pancreatic cells [5•] and to increase the expression of GLUT4, which is mostly found on skeletal muscle cells [3]. SCFAs are also a factor in activating AMPKs, which promote glucose transport and inhibit glycogen synthesis in skeletal muscle tissue. In the liver, activated AMPKs lead to the decreased gene expression of gluconeogenic enzymes [2, 11].

#### Lipid Metabolism

SCFAs are substrates for long fatty acid (FA) synthesis. Acetate and butyrate can be used as a substrate to obtain acetyl-CoA, which can be used as a substrate for the tricarboxylic acid cycle or can be used for the synthesis of palmitate and stearate [3, 5•].

What is more, SCFAs take part in adipogenesis promotion. Studies on preadipocyte cultures show that the addition of acetate, propionate, and butyrate is associated with the raised expression of proteins such as FA binding protein 4 (FABP4) and FA transporter protein (FATP), and enzymes such as lipoprotein lipase (LPL) and FA synthase (FAS). All of the above are involved in lipid metabolism [5•], whereas through FFAR2 or PPAR-γ activation, acetate and propionate promote the conversion of preadipocyte to adipocytes [11]. Moreover, SCFAs block lipid accumulation in adipocytes stimulated by insulin. Thus, smaller and more responsive adipocytes are formed [12]. However, in adipose tissue, lipolysis is strongly repressed by SCFAs. The inhibition of lipolysis is mediated by FFAR2 via hormone-sensitive lipase (HSL) and protein kinase A (PKA) inactivation [2, 3].

It is also shown that by activating AMPKs, SCFAs increase FA oxidation in both the liver and muscle tissue and simultaneously inhibit de novo synthesis and lipolysis. In effect, the concentration of FAs in serum decreases, as well as body weight [2].

Besides the effect on FA metabolism, SCFAs are thought to have an impact on cholesterol concentration in serum. It is shown that propionate decreases cholesterol synthesis, whereas acetate, propionate, and butyrate are thought to enhance cholesterol uptake by the liver [2, 11].

#### Neuro-immuno-endocrine Regulation

SCFAs are thought to be part of the microbiota-gut-brain connection; the three most abundant SCFAs are detectable in cerebrospinal fluid, typically in the range of 0–171 μM for acetate, 0–6 μM for propionate, and 0–2.8 μM for butyrate. Moreover, SCFAs can cross the blood–brain barrier and affect its properties: improve integrity and reduce the permeability of the barrier. In the central nervous system, SCFAs influence brain cells such as neurons, astrocytes, and microglia, and their functions. Additionally, SCFAs interact with their receptors on enteroendocrine cells, promoting indirect signaling to the brain via systemic circulation or vagal pathways by inducing the secretion of γ-aminobutyric acid (GABA) and serotonin. SCFAs occurring in physiological concentrations increase the growth rate and intensify the mitosis of nerve cells. The impact of SCFAs on processes in the central nervous system may cause an increase in neurogenesis, and an improvement in cognitive development and memory. The addition of acetate and butyrate to microglial cells and astrocytes has been described and, as a result, inflammatory signaling was inhibited. Diseases indicated to be associated with SCFA alterations include neurodegenerative Alzheimer’s Disease and Parkinson’s Disease, but also Autism Spectrum Disorder, Multiple Sclerosis, and Major Depressive Disorder [20, 21].

#### Immunity

Obesity is shown to be related with chronic inflammation. Through GPRs, SCFAs can take part in modulating the immune response via modifying the release of cytokines including IL-18, an important factor in repairing the epithelial barrier in the gut. SCFAs can also regulate the differentiation, recruitment, and activation of immune cells: neutrophils, T lymphocytes, dendritic cells (DCs), macrophages. Moreover, SCFAs are shown to have anti-inflammatory effects by reducing some pro-inflammatory cytokines such as TNF-α and IL-12, affecting the capture of antigens by macrophages and DCs, and stimulating T lymphocytes [1, 22]. In turn, it is also shown that TNF-α, released from the anti-inflammatory macrophage M2-type, can take part in lowering the GLUT4 expression and, in effect, inhibit adipose tissue accumulation [8].

#### Anti-cancer Properties

SCFAs can have an impact on tumor cells. It is shown that propionate and butyrate can inhibit cell growth, thereby proliferating and invading cancer cells [23]. Interestingly, butyrate affects cells differently depending on its concentration. At a low concentration, butyrate supports healthy cells as an energy source, but when the concentration is increased butyrate induces cell cycle arrest and apoptosis in a p53-dependent manner. Furthermore, at a concentration of 0.5 mM or higher, butyrate increases the expression of anti-metastatic genes and inhibits the activation of pre-metastatic genes. This dual role is known as the “butyrate paradox” [4, 11, 23]. SCFAs are involved in the occurrence and progression of cancer through various mechanisms. Modulating HDACs lead to hindered cell attachment, the immigration of immune cells, stimulated cytokine production, chemotaxis, and apoptosis [24].

#### Anti-inflammatory Properties

Besides stimulating immune cells, as described above, SCFAs can also participate in inflammation regulation via transducing cell signals by binding to GPCRs or HDACs. As a result, SCFAs can reduce the production of pro-inflammatory cytokines and decrease oxidative stress. Due to activating FFAR2 or FFAR3 on macrophages and neutrophils, SCFAs decrease the expression of IL-8. Moreover, by binding to those receptors, butyrate down-regulates the levels of tumor necrosis factor (TNF), IL-6, and nitric oxide synthase (NOS). Butyrate is described to inhibit HDA. Therefore, SCFAs, as FFAR2 and FFAR3 agonists, may be potentially effective drugs for the treatment of inflammatory diseases [23].

#### Pro-inflammatory Properties

At present, SCFAs have been proved to be involved in regulating the host immune system, specifically regulating the differentiation and recruitment of immune cells, which are mainly mediated by G protein–coupled receptors on the cell membrane of immune cells. In the inflammatory response, SCFAs exhibit two opposite effects, anti-inflammatory and pro-inflammatory. It is reported that acetate can increase the production of pro-inflammatory cytokines such as IL-6, CXCL1, and CXCL2. Based on existing evidence, it is speculated that the conflicting results exhibited between pro-inflammatory and anti-inflammatory effects may be related to the local concentration of SCFAs, the carbon chain length, and the activated receptors [23].

In view of the prominent role of SCFAs in immunity and inflammation, the supplementation of SCFAs may be an effective strategy for the future treatment of inflammatory and immune-related diseases, such as rheumatoid arthritis. However, current research on SCFAs is too broad; thus, in-depth and detailed research is still required.

## SCFAs in Clinical Trials

SCFAs, due to their wide use as signaling molecules, have great potential against many diseases. Recently, many randomized controlled trials (RCTs) have been conducted examining the effect of butyrate administration on the development of various diseases. Butyrate supplementation may be beneficial for adult patients with inflammatory bowel disease, irritable bowel syndrome, ulcerative colitis, or Behçet’s Syndrome, but also for children with obesity. Butyrate administration was found to be ineffective in type 1 and type 2 diabetes. The available RCT results are summarized in Table 3.

**Table 3:** RCT results on butyrate administration

**Condition**	***n***	**Dose**	**Administration**	**Time**	**Results**	**Ref**
Inflammatory bowel disease	49	1.8 g/day	Oral	2 months	Increased the growth of bacteria able to produce SCFAs with potentially anti-inflammatory action	[25]
Pediatric obesity	54	20 mg/kg body weight	Oral	6 months	Greater changes in BMI, waist circumference, insulin level, ghrelin level, HOMA-IR, micro-RNA221 relative expression, and IL-6 level	[26]
Behçet’s Syndrome	17	2.4 g/day	Oral	3 months	Reduced leukocyte ROS production and lipid peroxidation in plasma, increased total antioxidant capacity in plasma, improvement in fibrin susceptibility to plasmin-induced lysis	[27]
Pediatric inflammatory bowel disease	72	300 mg/day	Oral	12 weeks	No difference in remission rate or median disease activity	[28]
Type 1 diabetes	30	4 g/day	Oral	1 month	No changes in innate or adaptive immunity in T1DM	[29]
Type 2 diabetes	39	100 mg/day	Oral	6 weeks	No changes in biochemical parameters	[30]
Irritable bowel syndrome	66	300 mg/day	Oral	12 weeks	Decreased frequency of spontaneous abdominal pain, postprandial abdominal pain, abdominal pain during defecation, stool consistency and constipation	[31]
Ulcerative colitis	16	6 mmol/day	Rectal	20 days	No changes in measured parameters of the colonic mucus layer: MUC2 and TFF3	[32]
Ulcerative colitis	11	100 mmol/day	Rectal	8 weeks	Reduced number of translocated NF-kB-positive macrophages, reduced number of neutrophils in crypt and surface epithelia and of the lamina propria lymphocytes/plasma cells, decreased Disease Activity Index (DAI)	[33]
Shigellosis	80	160 mmol/day	Rectal	3 days	Early reduction of macrophages, pus cells, IL-8 and IL-1β in the stool, induced LL-37 expression in the rectal epithelia	[34]

## SCFAs in Obesity/the Control of Obesity

Obesity is a complex condition caused by a variety of genetic and non-genetic factors. According to the World Health Organization, obesity is having a body mass index (BMI) greater than 30; however, the definition is different in some countries: in China, a BMI of 28 or greater is considered obese. The prevalence of obesity is rising worldwide. It is predicted that 1.12 billion people will be obese in 2030. Obesity increases the risk of diseases such as hypertension, dyslipidemia, insulin resistance (IR), glucose intolerance, type 2 diabetes, coronary heart disease, arthritis, sleep apnea, and some cancers [35, 36]. Moreover, it is associated with alterations in gut microbiota and the SCFA profile [37].

It has been repeatedly confirmed that obese patients have an increased total content of fecal SCFAs in comparison to lean patients [10, 38, 39]. Moreover, the composition of SCFAs changes. The percentage of propionate in the total concentration of SCFAs was higher in obese and overweight patients’ feces than in that of lean patients [38]. Additionally, later studies confirmed changes in fecal SCFAs related to obesity and adiposity parameters. It was shown that subjects with a higher concentration of butyrate have lower gut microbiota diversity, higher gut permeability, systemic inflammation (shown as a high level of highly sensitive C-reactive protein, hs-CRP), glycemia (higher fasting glucose levels), dyslipidemia, obesity (higher BMI), central obesity (higher waist circumference or visceral adipose tissue), and also hypertension [10, 40]. Rahat-Rozenbloom et al. considered the possible influence of dietary intake or the level of SCFA absorption on the variation between lean and obese/overweight patients. As a result, obese/overweight subjects had higher fecal SCFAs than lean subjects, with no difference in rectal SCFA absorption and with comparable food intake [41]. Coherent results came from a study of Fernandes et al. The researchers confirmed higher individual and total levels of SCFAs in the stools of obese/overweight patients in comparison to lean patients. What is more, there were no differences in dietary intake and the level of physical activity between these two groups [42•].

SCFAs are thought to be a preventive factor against obesity. It is shown that the supplementation of acetate, propionate, or butyrate to a high fiber diet can inhibit body weight gain in diet-induced obese mice, and influence biochemical parameters in serum, such as triglycerides or cholesterol. The colonic mRNA expression of both FFAR2 and FFAR3 is raised following obesity. The dietary addition of SCFAs leads to changes in the gene expression of GPRs: an increase in the adipose tissue and a decrease in the colon. Other effects described in the study are higher PYY, GLP-1, and leptin secretion, enhanced TG hydrolysis and FFA oxidation in the adipose tissue, and inhibited chronic inflammation [43]. The studies conducted so far also indicate the inhibition of lipolysis and adiposity, as well as an improvement in gut integrity, an increase in energy expenditure, and thermogenesis. The effects of SCFA administration in in vitro and in vivo models supporting the role of SCFAs in the treatment of obesity are summarized in Table 4.

**Table 4:** Results of studies on the administration of SCFAs in obesity and obesity-related diseases

**Administration**	**Model**	**Effect on obesity control**	**Ref.**
Isobutyrate, isovalerate	Primary adipocytes	↓Lipolysis, ↓lipogenesis, ↑insulin-stimulated glucose uptake	[44]
Acetate, propionate, butyrate	NCI-h716 and HuTu-80 cells	↑PYY expression	[16]
Butyrate	cdx2-IEC cells	↑Gut barrier integrity	[45]
Butyrate	Caco-2 cells	↑Gut barrier integrity	[46]
Butyrate	Lamina propria cells and peripheral blood cells	↓Inflammation	[47]
Propionate, butyrate	Caco-2/TC-7 cells	↓Cholesterol biosynthesis	[48]
Butyrate	Caco-2 cells	↓Chylomicrons and ↓very low-density lipoprotein secretion	[49]
Acetate, butyrate	Rat colon	↑PYY and GLP-1 secretion	[50]
Butyrate	Mice and RAW 264.7 macrophages	↓Atherosclerosis	[51]
Propionate	Human colonic cells	↑PYY and GLP-1 secretion, ↑energy expenditure	[52]
Acetate, propionate, butyrate	C57BL/6 J mice	↓Body weight gain, ↓plasma free FAs, ↑GPR43 expression in adipose tissue, ↑adiponectin and resistin expression, ↑fat oxidation, ↑microbiota richness	[43]
Butyrate	C57BL/6 J mice	↓Inflammation	[53]
Butyrate	C57BL/6 J mice	↓Body weight gain, ↓inflammation, ↑gut barrier integrity	[54]
Butyrate	C57BL/6 J mice	↓Body weight gain, ↓adiposity	[55]
Butyrate	C57BL/6 J mice	↑Insulin sensitivity, ↑energy expenditure, ↑FA oxidation, ↑thermogenesis, ↓adiposity	[56]
Butyrate	C57BL/6 J mice	↑Thermogenesis	[57]
Acetate, butyrate, propionate	C57BL/6N mice	↑PYY and GLP-1 secretion	[58]
Butyrate	C57BL/6 J mice	↓Body weight gain, ↓fat deposition	[59]
Butyrate	APOE*3-Leiden.CETP mice	↓Energy intake, ↑fat oxidation	[60]
Acetate, propionate, butyrate	C57B/6 mice and 129/SvEv mice	↑GLP-1 secretion	[61]
Butyrate	C57BL/6 J mice	↓Inflammation, ↓fat deposition	[62]
Acetate, propionate, butyrate	Wistar-ST rats	↓Plasma cholesterol	[63]
Acetate	Human	↑Fat oxidation, ↑PYY secretion, ↓inflammation, ↑postprandial insulin secretion	[64]
Butyrate	Human	↓Inflammation, ↑antioxidant responses	[65]
Acetate, propionate, butyrate	Human	↓Lipolysis, ↑fat oxidative capacity, ↑PYY secretion	[66]

There can be several mechanisms explaining high fecal concentrations of individual or total SCFAs: increased microbial production, shifts in colonic cross-feeding patterns, fluctuations in mucosal absorption, or the rate of transit alone [38].

However, other studies indicated that similar effects cannot be observed in serum or plasma samples where circulating propionate and butyrate levels may be constant or decreased in patients with a higher BMI compared to those with a lower BMI [10, 67•]. However, the butyrate/isobutyrate ratio, isovalerate, and total SCFA levels in plasma were shown to be positively associated with the weight/height ratio; and the butyrate/isobutyrate ratio was positively associated with the BMI as well [67•]. In addition, a positive correlation between circulating acetate, propionate, and butyrate concentrations and a fasting GLP-1 concentration has also been described. Moreover, the same studies indicate that the plasma level of SCFAs is associated with the BMI but the fecal level of SCFAs is not related to the BMI, which is in contradiction to previous research [68].

Information on changes in the blood concentrations of SCFAs in the development of obesity is incomplete. Feces are often the material used for testing due to the higher concentrations of these metabolites, which facilitates measurement. However, further research is needed, especially since only about 5% of all SCFAs are excreted and the rest are absorbed into the bloodstream.

**Fig. 3:**
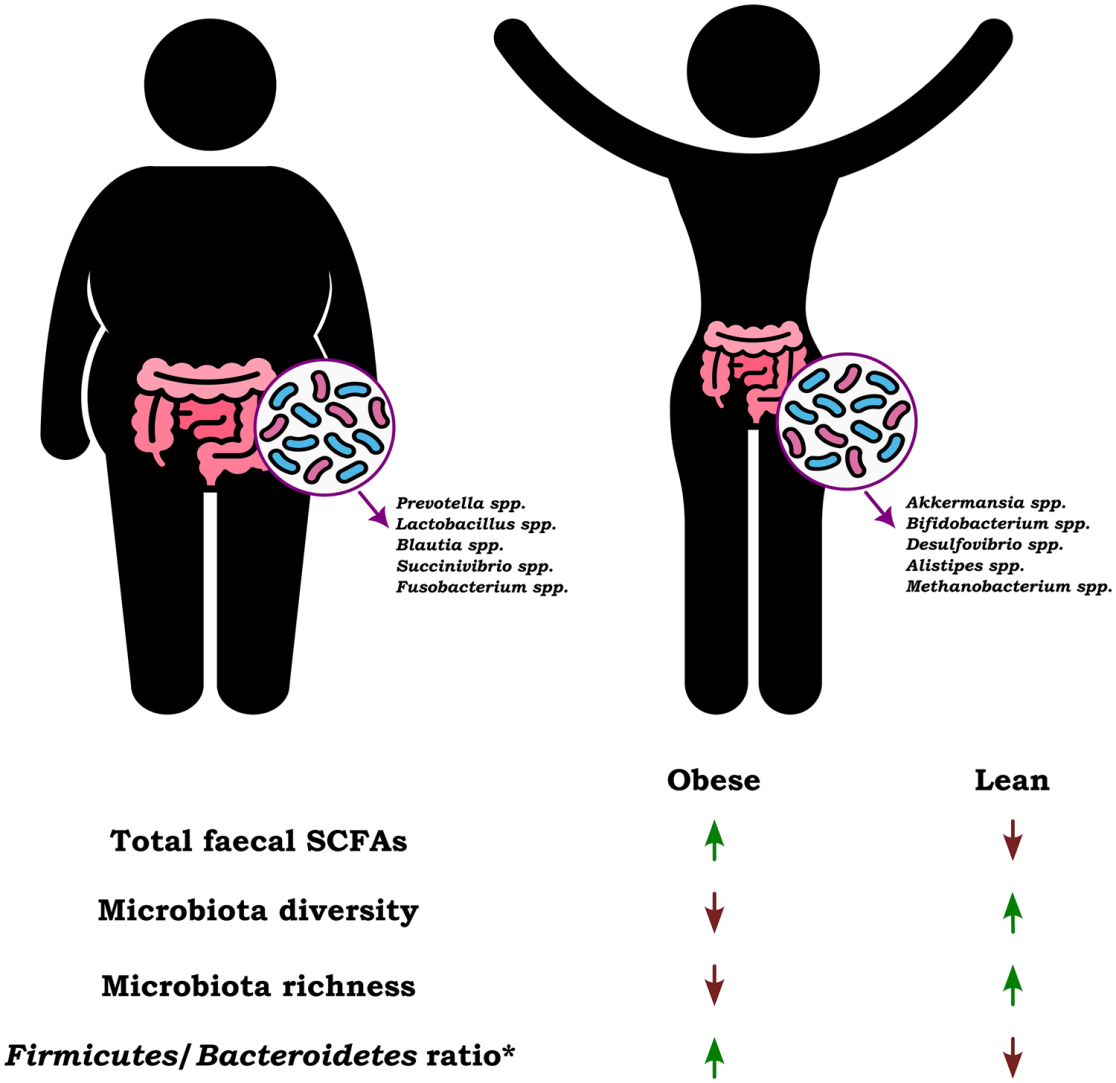
Summarized differences between obese and lean individuals regarding total SCFAs in feces, microbiota diversity, specific microbiota, and the *Firmicutes/Bacteroidetes* ratio [36, 72]. *Most sources indicate an increase in the *Firmicutes/Bacteroidetes* ratio in obese patients compared to lean individuals [37, 73, 74•]. However, there are publications that describe the opposite results [38, 72]. The utility of this indicator is rather unclear [70]. “↑” – higher concentration, “↓” – lower concentration

Besides SCFAs, shifts in gut microbiota are also proved to be relevant in obesity. *Firmicutes* and *Bacteroidetes* are the most abundant in the feces of both obese and lean groups [38, 69]. The *Firmicutes*/*Bacteroidetes* ratio was previously considered an appropriate microbiota marker that could indicate dysbiosis and microbiota imbalances, but due to inconclusive results, its relevance is uncertain [70–72]. Some studies confirmed a higher percentage of *Firmicutes* in obese/overweight patients [37, 73, 74•], but others found no significant difference in *Firmicutes* and even higher relative levels of *Bacteroidetes* [38]. Another important factor related to obesity is the diversity of the gut microbiota. Most studies have shown that the diversity and richness of the gut microbiome are reduced in obese subjects [36, 69]. The differences between obese and lean groups are presented in Fig. 3.

On the other hand, some studies suggest that in obesity, changed gut microbiota can produce more SCFAs, and also more energy from the diet than lean individuals, and thus be partly causative [42•, 69]. These conclusions are based on an animal study. In humans, it is most likely that obesity-related microbiota are associated with dietary intake rather than being a cause of this condition [73].

### SCFAs and Diet

SCFAs are a product of dietary carbohydrate fermentation. Fiber can be divided into polysaccharides, oligosaccharides, and resistant starches, due to the structure of monomers. Changes in dietary fibers can impact the composition of gut bacteria. High dietary fiber consumption is associated with increased gut microbiota diversity and lower long-term weight gain [2, 75]. It is also linked to a reduced risk of obesity, mostly due to SCFA-related modulation of gut hormone secretion [42•]. Therefore, the supplementation of fibers may be valuable in obesity treatment and lead to increased richness and diversity of microbiota, and also SCFA production [76]. Moreover, supplementation with prebiotics will affect the production of SCFAs. It has been described that the intake of prebiotic inulin in obese and overweight men leads to a significant increase in the plasma concentration of acetate, as well as a trend towards higher plasma butyrate concentrations when compared with the placebo group. Increased fat oxidation and a decrease in glucose and insulin concentrations were also reported [77].

Sowah et al. described the effects of dietary weight loss interventions. Calorie-restricted diets for obese or overweight patients can reduce the concentration of total SCFAs in feces or not change it significantly. Weight loss as a result of following a diet decreases primarily butyrate levels. The most pronounced changes among SCFAs were observed with low-carbohydrate diets; however, in children, a decrease in the level of SCFAs was also observed despite the maintenance of a standard amount of carbohydrates in the diet [78]. Moreover, a comparison of the influence of low-calorie Mediterranean and vegetarian diets on the SCFA profile does not indicate statistically significant differences [79]. In turn, there are very few data on serum or plasma levels of SCFAs in response to diet and weight loss. It is shown that a calorie-restricted diet causes a lower acetate concentration in serum, but conclusions on other acids are lacking as their concentrations were not analyzed in this study [80]. The downward trend in SCFA concentrations after weight loss may indicate reduced efficiency in obtaining energy from dietary SCFAs, which are reportedly increased in obese individuals. An additional explanation is the increased mucosal absorption and the use of SCFAs in peripheral tissues and colonocytes in response to prolonged caloric restriction [78]. However, further extensive studies on the effects of diet on obese patients and their SCFA profiles are needed as the outcomes from current studies are inconclusive.

## Bariatric Surgery

Due to the high prevalence of obesity, there is a need for more effective treatment. Pharmacotherapy and diet medications are inefficient. Still bariatric surgery (BS) seems to be the most effective treatment, providing long-term weight control and the resolution or improvement of comorbidities, leading to a better quality of life [81]. BS works through various mechanisms. Above all, it reduces the digestion of nutrients, alters food preferences, accelerates gastric emptying, and regulates hormonal changes. One of the mechanisms involved in the action of BS is the change of microbiota and bile acid profiles [81, 82]. BS, despite the fact that it is an invasive therapeutic method, is considered to be safe and associated with a potentially small number of complications. Thanks to standardized protocols for postoperative care, the percentage of observed nutritional deficiencies is relatively low compared to periods in the past. Some complications observed at the early and late stages include gastric obstruction, gastroesophageal reflux, or internal herniation [82] as well as apnea and cardiopulmonary arrest, atelectasis, pulmonary embolism, anastomotic bleeding, rhabdomyolysis, anastomotic stenosis of the gastrointestinal tract, marginal ulcers, Wernicke encephalopathy, peripheral neuropathies, metabolic bone disease, or gallstones; however, they are relatively rare [35]. Nutritional deficits are more common. Patients after BS can develop iron, folate, vitamin B12, vitamin D, and calcium deficiencies [83]. The risk of micronutrient malnutrition may be a result of poor diet quality, weight loss advised before BS, decreased sun exposure, or medication side effects. Obesity is often connected with anemia of chronic disease: chronic inflammation promotes hepcidin synthesis, which leads to decreased iron absorption into the bloodstream. In addition, the level of micronutrients is influenced by the type of procedure and possible postoperative complications such as nausea, vomiting, or food intolerances. Taking everything into consideration, there is a strong recommendation for micro- and macronutrient supplementation after BS [84].

**Fig. 4:**
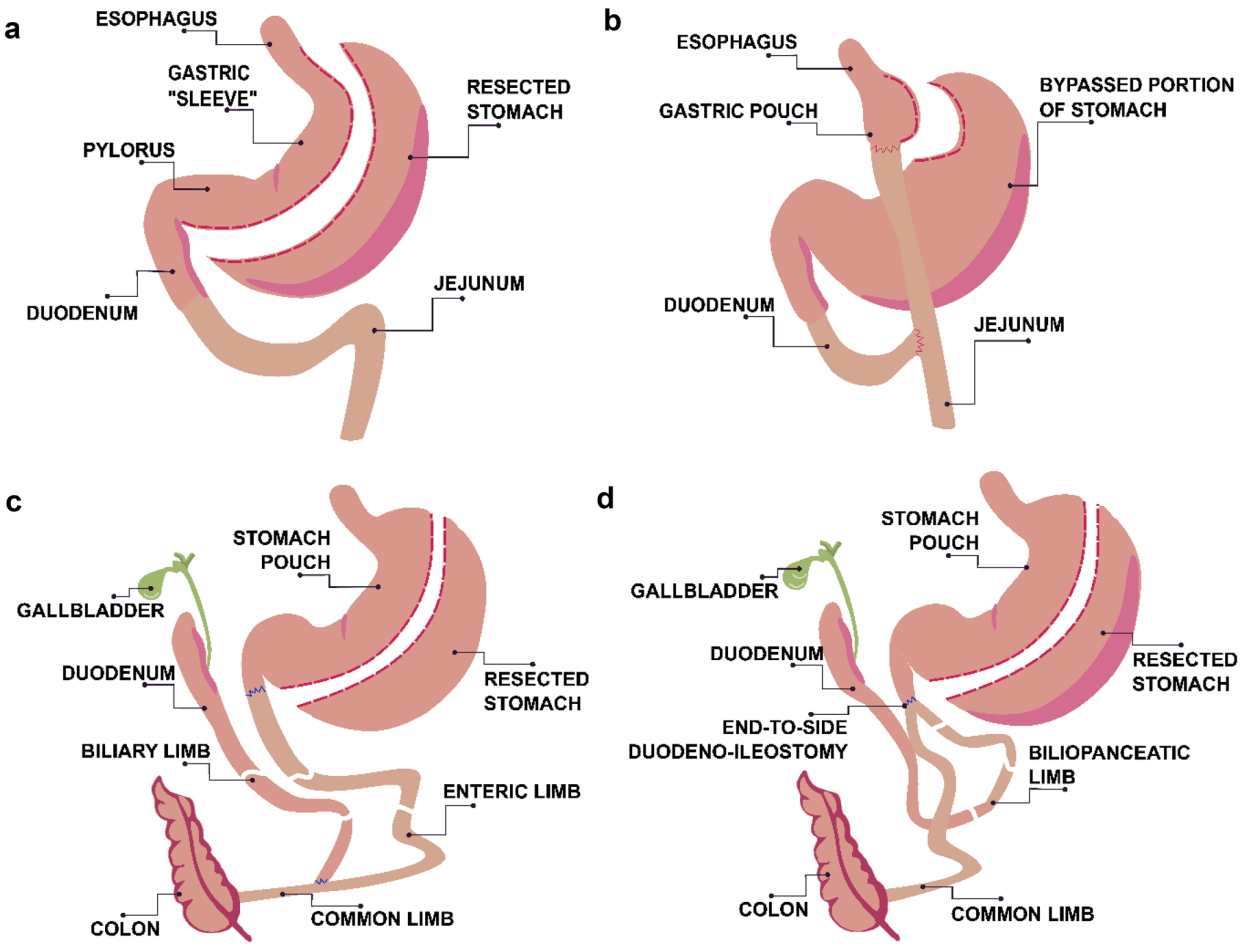
Different types of BS: **a** sleeve gastrectomy (SG), **b** Roux-en-Y gastric bypass (RYGB), **c** biliopancreatic diversion with duodenal switch (BPD/DS), **d** single anastomosis duodeno-ileal bypass with sleeve gastrectomy (SADI-S)

Among BS, there are three groups of procedures: restrictive, malabsorptive, and combined restrictive and malabsorptive procedures. Restrictive procedures lead to a decrease in the size of the stomach leading to a smaller intake of solids; malabsorptive ones shorten the small intestine and then lower the absorption of the nutrients. Schematic diagrams of four different procedures are shown below: one restrictive (sleeve gastrectomy, SG), and three combined (Roux-en-Y gastric bypass, RYGB; biliopancreatic diversion with duodenal switch, BPD/DS; and single anastomosis duodeno-ileal bypass with sleeve gastrectomy, SADI-S) (Fig. 4).

Sleeve gastrectomy (SG) is a laparoscopic procedure which leads to reducing the size of the stomach by resecting a large portion (about 80%) of the greater curvature. In effect, a narrow tube or sleeve is created. The resection originates from the antrum and runs up to the cardia portions of the stomach. This is an optimal procedure for extremely obese patients and young patients. Nowadays considered to be the most often performed operation worldwide, Roux-en-Y gastric bypass (RYGB) is the most common used type of BS, in which a 15–30-cm-long pouch stemming from the proximal stomach is connected to a loop of the jejunum. As a result, an anastomosis between the stomach and the proximal part of the jejunum is created. The remaining part of the stomach and the proximal small bowel are re-anastomosed 80–120 cm distal to the stomach and jejunum anastomosis, which allows the nutrient to flow [85, 86]. Biliopancreatic diversion with duodenal switch (BPD/DS) is a procedure whereby a portion of the stomach is resected, and a distal portion of the small intestine is connected to the remaining gastric pouch, bypassing the duodenum and jejunum. This approach results in the greatest weight loss, but, on the other hand, it is also associated with many disadvantages because of nutritional complications [82, 85, 86]. Single anastomosis duodeno-ileal bypass with sleeve gastrectomy (SADI-S) is a modification of BPD/DS with fewer anastomoses. It consists of biliopancreatic diversion and sleeve gastrectomy followed by an end-to-side duodeno-ileal diversion with an omega loop of the ileum. This approach eliminates jejunoileal anastomosis, which decreases the risk of complications [87]. It is worth mentioning older techniques such as jejunoileal bypass (JIB) and biliointestinal bypass (BIB), and single anastomosis duodenal jejunal bypass (DJB-sa). Both JIB and BIB construct a bypass to most of the small intestine and absorptive surfaces. JIB uses anastomosis between the jejunum and ileum, and BIB between the beginning and end-part of the small bowel, and connecting the disabled small bowel sling to the gallbladder [88]. DJB-sa requires anastomosis of the duodenum and jejunum. It does not change the diversion of the biliopancreatic flow [89].

## SCFAs After BS in Human Studies

BS directly influences the digestive tract and improves metabolism by affecting many hormones and receptors. The effects include weight loss, improved glucose metabolism, decreased adiposity and cholesterol serum level, lower energy intake, and inhibited inflammation. The exact BS impact on the gut bacteria and the production of SCFAs is still a field for research. The effects of RYGB are most often described, as it is the most frequently performed procedure [90]. The study of Salazar et al. denotes a decreased fecal concentration of acetate 1 month and 3 months after the procedure compared to time before the surgery. Other SCFAs did not achieve any significant change, aside from isobutyrate and isovalerate, which are increased already 3 months post-surgery. Raised fecal levels of acetate, as well as propionate and butyrate 4 months and 6 months after BS, are described by Meijer et al. and Farup et al., respectively [74•, 91]. Profiles of fecal straight SCFAs more than 9 months after RYGB are not altered in comparison to time before the procedure; however, levels of BSCFAs remain elevated [92, 93]. The improvement in health as a result of the operation is evidenced by the normalization of biochemical parameters in blood such as C-reactive protein (CRP), hemoglobin A1c (HbA1c) or zonulin, which is protein modulator of intercellular tight junctions and a marker of gastrointestinal permeability [74•].

Considering other types of surgeries, although Salazar et al. did not find any decrease in SCFA concentrations in the feces of SG patients 1 and 3 months after surgery compared to the time before SG [70], Meijer et al. observes such changes 4 months after and Farup et al. 6 months after SG. The authors indicate that there is no representative variation between the effects of RYGB and SG regarding the fecal SCFA profile nor the proportion of SCFAs and BSCFAs [74•, 91]. RYGB as a combined procedure, besides restrictive purposes, rearranges the gastrointestinal tract, which alters pH, oxygen content, bile acid concentration, and nutrient exposure in the colon, which can stimulate microbial diversity [92]. Although the differences between the effectiveness of RYGB and SG have not been confirmed so far, due to the different anatomical rearrangement, this is an area for further research.

One of the many results of BS and reduced dietary intake is a shift in gut microbiota, which, along with decreased SCFA and increased BSCFA concentrations in feces, is a sign of reduced saccharolytic in favor of proteolytic fermentation. BS affects satiety and lowers carbohydrate intake as well as the absorption capacity of the digestive tract, which results in less fiber and more protein and amino acids in the large intestine [74•]. Furthermore, the level of BSCFAs in feces shows a positive correlation with a high protein diet, and a negative correlation with high fiber consumption [7]. Knowledge about the function of BSCFAs in the body is not extensive. In vitro models suggest that BSCFAs may affect glucose and lipid metabolism [44]. However, understanding the importance of BSCFAs seems to be valid because many of the studies described above indicate their increase in the colon shortly after surgery.

In addition to changes in BSCFAs, the shift of metabolic pathways in bacteria after BS is evidenced by altered propionate/acetate and butyrate/acetate ratios in the feces, which are higher in patients after RYGB. Enriched microbiota and conditions after BS favor the accumulation of more reduced SCFAs such as butyrate, rather than acetate [92, 93]. Propionate and butyrate are involved, among others, in glucose and lipid metabolism as signaling molecules for multiple receptors. In conclusion, BS decreases the concentration of fecal SCFAs but increases that of BSCFAs, as well as changing the proportion among them, which can affect the outcome of surgery related to weight loss and the improvement of health.

Few studies on the long-term effects of BS have been conducted. The study of Juárez-Fernández et al. describes the impact of BS after 4 years. The fecal SCFA profile compared to the pre-BS profile is altered, and all the acetate, propionate, and butyrate levels are decreased. However, this study has limitations, such as a small sample size, and the impact of the type of operation was not specified [94]. Nevertheless, observations made by Tremaroli et al. on patients after 9 years post-BS describe a slight tendency for fecal SCFAs to decrease and a slight tendency for BSCFAs to increase after RYGB, but with no statistical significance. In turn, a lowered fecal SCFAs/BSCFAs ratio achieved significance for RYGB patients [95]. Both papers suggest an abundant impact on metabolism and long-lasting effects regarding the SCFA profile. Obesity is connected with higher levels of SCFAs, while BS mainly with lowering SCFA levels. However, according to Ilhan et al., after 9 or more months post-surgery, there is still a significant difference between patients after BS and lean patients [92]. The long-term effects of the operation also include a significant decrease in biochemical parameters in blood, such as fasting glucose, fasting insulin, CRP, and HbA1c [94].

What is more, BS implies drastic changes in dietary intake. Interestingly, comparing SG and a very low-calorie diet (800 kcal obtained by prepared sachets), there are no significant changes in the fecal SCFA profile after 3 and 6 months, despite observed changes in the composition of the microbiome and microbial capacity for butyrate fermentation [96]. The authors indicate the reasons for such results in the low fiber content and lack of prebiotics in the diet. Thus, the impact of a restrictive diet on the results obtained after surgery cannot be excluded.

Unfortunately, the effect of BS on the SCFA profile in the blood is poorly understood. Information related to changes in concentrations or proportions of circulating SCFAs is lacking. It has been shown that alterations observed in plasma are not necessarily reflected in stool samples since only a small percentage of SCFAs are excreted. What is more, as described above, although a positive correlation between fecal butyrate levels and the BMI has previously been reported, the circulating concentrations of butyrate and propionate were inversely associated with the BMI [10, 68]. This confirms that the fecal profile of SCFAs provides only a partial understanding of the impact of obesity and BS on the body, and therefore further research is needed.

BS significantly interferes with the structure of the digestive system and affects the composition of the microbiome and the concentration of its metabolites, primarily SCFAs. These metabolites are involved in the regulation of many systems, such as glucose and lipid metabolism, satiety control, immunity, gut integrity, blood pressure regulation, or nervous system maintenance. Obesity is a growing problem in modern society, which is why the importance of BS is also increasing. Not only the surgery itself, but also a change in eating habits afterwards is important for the microbiome. A better understanding of the relationship between the patient and the microbiota metabolome after different types of BS may allow for a more accurate selection of patients for different procedures and may lead to the development of personalized prebiotic and dietary interventions for the optimal impact of BS on the health of patients.

### Challenges of Studies on SCFAs in Humans

Information on the content of SCFAs in the body of both healthy and obese people is still only partial. Research on this topic is undoubtedly a challenge due to the following reasons:

- Habitual food choice – SCFAs are produced by intestinal bacteria in response to prebiotic intake, including various types of fiber. However, prebiotic intake leads to differential effects on SCFA production in human studies, and the responses of gut microbes to nutritional interventions may vary from person to person [97].
- Ethnicity and geography – The composition, diversity, and activity of intestinal microbiota, and at the same time, the composition of SCFAs, can be easily modified by dietary patterns or specific nutrients, which differ between continents. In addition, geographical location, ethnicity, and the level of urbanization also have an impact on gut microbiota [72, 98, 99].
- Follow-up – Long-term studies related to SCFAs, obesity, and the impact of bariatric surgery are lacking. Such studies are expensive, and recruiting patients and collecting large amounts of data is difficult.
- Encouraging people to include fiber in their diet – The Western diet is characterized by a lower supply of fiber compared to diets rich in vegetables and fruits, which affects the level of SCFAs produced. However, persuading people to change their diet and include fermentable fiber is still a challenge [100].
- Individual variability – Variability in the baseline microbiota of participants is also a difficulty in human SCFA studies [11]. Not every patient responds to the given prebiotic, or the reactions may differ from each other [97].
- Lack of access to the appropriate matrix – The in vivo SCFA production rates as well as the intestinal SCFA concentrations on different fibers are most relevant in SCFA studies. However, the measurement of the cecal SCFA concentration is almost impossible due to the inaccessibility of the colon and rapid absorption by the colonocytes. Usually conclusions about cecal and colonic metabolism are deduced from fecal content and in vitro studies. Human colonic in vitro gut models are widely used to estimate levels and ratios of SCFA production from different substrates, but they cannot account for in vivo absorption [2, 101].
- Gut microbiota capacity – The stimulation of SCFA production will not always be effective due to the fact that the gut microbiota of individuals is limited in their overall capacity to produce fecal SCFAs from fiber [97].
- Translating results from animal studies – Translating the effects of SCFAs from animal to human studies is limited by physiological and dietary differences. The delivery of a sufficient amount of SCFAs based on results on animal models to target sites that include the colon and the systemic circulation is also a limitation [100].
- Examined group – Usually the industrial population is included in study groups. It seems to be important to include also non-industrial individuals due to different lifestyles which influence gut microbiota and SCFA production [99].
- Low concentration in blood serum or plasma – SCFAs from the colon are largely absorbed into the colonocytes. Then, they are used as an energy source and are immediately oxidized. The remaining SCFAs are transported to the liver via the portal circulation where another fraction is metabolized. Only the SCFAs that pass the liver and escape splanchnic extraction end up in the peripheral circulation [102].
- Blood tube choice – Blood concentrations of SCFAs are relatively low and can be affected by the selection of the test tube. SCFA serum should be collected in a tube without additives and without a separating gel, as they may lead to contamination and falsely higher concentrations. In turn, plasma should be collected in a heparin tube. An EDTA tube causes high acetate levels [102].
- Methodological difficulties – During sample preparation, there are several steps that can generate errors. Extraction with ethyl acetate in an acidic environment can increase the acetate level in the sample. The derivatization step should be carried out under anhydrous conditions; otherwise, SCFA losses may occur. It is easy to contaminate samples with acetate, e.g., from test tubes or plastic tips. The limit of detection (LOD) varies between the methods used, especially between methods with and without a derivatization step. The LOD is also lower in GC–MS compared to GC-FID. Thus, the derivatization step increases the sensitivity of the analysis [103].

## SCFAs After BS in Animal Models

Undoubtedly, BS leads to a shift in gut microbiota and their metabolites, including SCFAs. Changes in fecal SCFAs described in animal studies are ambiguous and differ from those obtained in patients (Table 5). There are a few studies on animal models concerning this issue. However, the results are inconclusive (Table 6).

**Table 5:** Summary of changes in the fecal SCFA profile in patients after BS

Surgery	Sample (*n*)	Time post-op (weeks/years)	Changes in SCFAs							Study
			Acetate	Propionate	Butyrate	Isobutyrate	Valerate	Isovalerate	Total SCFA	
	26	1w	**↓**	**↔**	**↔**	**↔**	**↔**	**↔**	**-**	[70]
	26	3w	**↓**	**↔**	**↔**	**↑**	**↔**	**↑**	**-**	[70]
RYGB	14	4w	**↓**	**↓**	**↓**	**-**	**↓**	**-**	**↓**	[91]
	73	6w	**↓**	**↓**	**↓**	**↑**	**↔**	**↑**	**↓**	[74•]
	7	6w	**↔**	**↔**	**↔**	**↑**	**↔**	**↑**	**↔**	[93]
	9	12w	**↔**	**↔**	**↔**	**↑**	**-**	**↑**	**-**	[93]
	14	1w	**↔**	**↔**	**↔**	**↔**	**↔**	**↔**	**-**	[70]
SG	14	3w	**↔**	**↔**	**↔**	**↔**	**↔**	**↔**	**-**	[70]
	8	4w	**↓**	**↓**	**↓**	**-**	**-**	**-**	**↓**	[91]
	17	6w	**↓**	**↓**	**↓**	**↑**	**-**	**↑**	**↓**	[74•]
SG/BDP/bypass	6/2/1	4y	**↓**	**↓**	**↓**	**-**	**-**	**-**	**-**	[94]

“↑” – higher concentration vs before BS, “↓” – lower concentration vs before BS, “↔“ – no significant changes vs before BS, “-” – no data

**Table 6:** Summary of changes in the fecal SCFA profile after BS in the animal model

Surgery	Sample (*n*)	Model	Time post-op (weeks)	Changes in SCFAs						Study
				Acetate	Propionate	Butyrate	Isobutyrate	Valerate	Isovalerate	
SG	7	Rats	3	**↔**	**↔**	**↔**	**↔**	**↔**	**↔**	[104]
	7		8	**↔**	**↔**	**↔**	**↔**	**↔**	**↔**	[104]
	11		1	**↓**	**↓**	**↔**	**-**	**↔**	**-**	[90]
	11		2	**↓**	**↓**	**↔**	**-**	**↔**	**-**	[90]
RYGB	7	Rats	3	**↔**	**↑**	**↔**	**↔**	**↑**	**↑**	[104]
	11		4	**↓**	**↓**	**↓**	**-**	**↓**	**-**	[90]
	7		8	**-**	**↑**	**↑**	**↔**	**↔**	**↔**	[104]
	6	Mice	2	**↓**	**↑**	**↔**	**-**	**-**	**-**	[107]
SADI-S	9	Rats	3	**↑**	**↑**	**↑**	**↑**	**↔**	**↑**	[104]
	9		8	**↔**	**↑**	**↑**	**↔**	**↔**	**↔**	[104]
BDP-DS	7	Rats	3	**↔**	**↑**	**↑**	**↑**	**↑**	**↑**	[104]
	7		8	**↔**	**↑**	**↑**	**↑**	**↔**	**↑**	[104]
DJB-SA	6	Rats	8	**↑**	**↑**	**↑**	**↑**	**↑**	**↑**	[89]

“↑” – higher concentration vs obese control, “↓” – lower concentration vs obese control, “↔“ – no significant changes vs obese control, “-” – no data

In general, various types of BS lead to shifts in gut microbiota and fecal SCFA concentrations simultaneously, usually with elevated levels of SCFAs [89, 104–106]. The study of Mukorako et al. compares the effects of procedures involving absorption limitations, such as RYGB, BDP-DS, SADI-S, and restrictive SG, in rats. In conclusion, hypoabsorptive procedures contribute to higher fecal levels of all SCFAs besides acetate in comparison to obese controls. In turn, after SG, there seems to be an increasing trend for fecal propionate and butyrate concentrations in time, but with no statistical significance [104]. Nevertheless, in another rat model, the concentrations of fecal acetate and butyrate are described to rise after SG in comparison to pair-fed controls. The study did not compare RYGB to an obese control. RYGB is also thought to influence SCFA receptors: mRNA and the protein expression of FFAR2 and FFAR3 in the ileum are elevated [106]. In turn, Peiris et al. found that only the mRNA expression of FFAR2 increases after RYGB compared to the obese group [105]. A complementary study performed on mice models confirms the impact of RYGB on the SCFA profile in feces. The level of acetate was described to be lower and the level of propionate to be higher after the RYGB procedure [107]. The obtained results regarding elevated propionate and butyrate over acetate are congruent with findings in humans, where RYGB is known to raise the propionate/acetate and butyrate/acetate ratios in stool samples [92]. Interestingly, also changes in BSCFAs in feces are shown. Increased levels of isobutyrate and isovalerate can be the result of a shift in microbiota and incomplete protein and amino acid digestion due to the specification of malabsorptive or combined surgeries [104]. Elevated levels of BSCFAs are also reported in patients after RYGB and SG [74•, 93].

On the other hand, Seyfried et al. reported contradictory results. The concentrations of acetate, propionate, butyrate, and valerate in feces in a group of rats after RYGB decreased compared to obese controls at all measured post-surgical time points. Due to the procedure, the gut microbiota is altered and may have lower fermentation activity. The bioavailability of fiber in the colon may be reduced as well. Furthermore, in this study, acetate was measured as the most abundant SCFA in blood. The concentration of acetate in plasma in the RYGB rat group is higher in comparison to both obese and pair-fed controls, but does not attain a statistical significance [90].

In addition, Yu et al. describes the results of fecal SCFA levels after DJB-SA. Rats’ feces after this procedure are characterized by elevated levels of all SCFAs including BSCFAs compared to time before surgery and to obese controls. Moreover, the intestinal expression of receptors such as FFAR2, FFAR3, and GPR109a is increased [89]. As a result of the changes described above, glucose metabolism may be altered. The researchers also indicate a positive correlation between the level of propionate, butyrate, isobutyrate, and isovalerate and PYY, which can indicate beneficial metabolic and energy homeostasis actions including lower levels of fasting blood glucose or fasting serum insulin [89, 104].

Knowing that BS leads to a reduction in food intake, the question is to what extent can restricted dietary intake contribute to these results? Recent studies, which concern the consequences of BS, indicate a diet-independent nature of postoperative changes. In a study by Mukorako et al., levels of fecal SCFAs and BSCFAs are similar between obese control and pair-fed control animals [104]. The profile of fecal SCFAs is remarkably altered between groups after various types of BS, and subjects with caloric restriction. Most of the studies denote a coherent alteration in SCFAs when it comes to comparing post-surgery vs obese control and post-surgery vs dietary-restricted pair-weighed control. This applies both to the affected SCFAs and time points after the procedure [104, 107]. However, a study by Seyfried et al. indicates that significant changes in SCFAs between post-RYGB and dietary-restricted groups occur at later time points, which can denote the partial relevance of limited consumption in the achieved metabolism improvement after BS [90].

The analysis of the effects of BS on the SCFA profile in feces is ambiguous, both in terms of the direction of changes, the affected acids, or the time of changes. All of the above animal studies have some limitations. There are obvious differences between humans, mice, and rats, although the rodent model has been proved to be accurate to evaluate the mechanisms and results of BS. The procedure in rodents is diverse, it is not performed laparoscopically, and no staples are used. Moreover, the lifespan and lifestyle are different, as well as the composition of the gut microbiome between these species. What is more, the sample size is often limited due to the complexity of the procedure of performing surgery on rats or mice. Therefore, conclusions from animal experiments could not be extrapolated to human settings, and it is necessary to perform more clinical studies in this area [104, 106].

## Conclusions

SCFAs, as bacteria metabolites, are proved to be an important part of human metabolism regulation. Due to their actions related to glucose and lipid metabolism, as well as appetite and satiety control, they are considered to be a factor helping in the control of obesity. The extension of knowledge of the role of SCFAs in obesity and BS may lead to an improved BS procedure through prebiotic and dietary interventions. Depending on the SCFA profile results in both feces and blood, a personalized probiotic and dietary intervention may be designed in future and utilized to take advantage of the potential of SCFAs against obesity-related diseases. An increased content of SCFAs in the feces of obese patients has been described many times; however, the significance of this phenomenon is less understood, as well as alterations in the SCFA profile after BS. The considered changes in fecal levels of SCFAs concern only a few percent of the SCFAs produced, the vast majority being absorbed into the circulation. Determining the relationship between changes in SCFA concentrations in the stool and in the blood of obese patients seems to be necessary.

Human studies on SCFAs are still a challenge due to difficulties in measuring SCFA production. Plasma and serum are rarely analyzed, probably due to low SCFA concentrations and a lack of standardization. Moreover, other limitations in human studies include variations in diet composition and metabolic phenotype between patients. However, further research is required, especially longer-term controlled studies to explore and understand the actions of SCFAs in obesity and metabolic health.
